# Supplementary material for: The Aqueous Extract of Brassica oleracea L. Exerts Phytotoxicity by Modulating H2O2 and O2− Levels, Antioxidant Enzyme Activity and Phytohormone Levels
Source: Plants (Basel). 2023 Aug 28;12(17):3086. doi: 10.3390/plants12173086 (PMC10490512; doi:10.3390/plants12173086)
Supplement: Supplementary file 1 [file plants-12-03086-s001.zip › plants-2523997-supplementary.pdf]

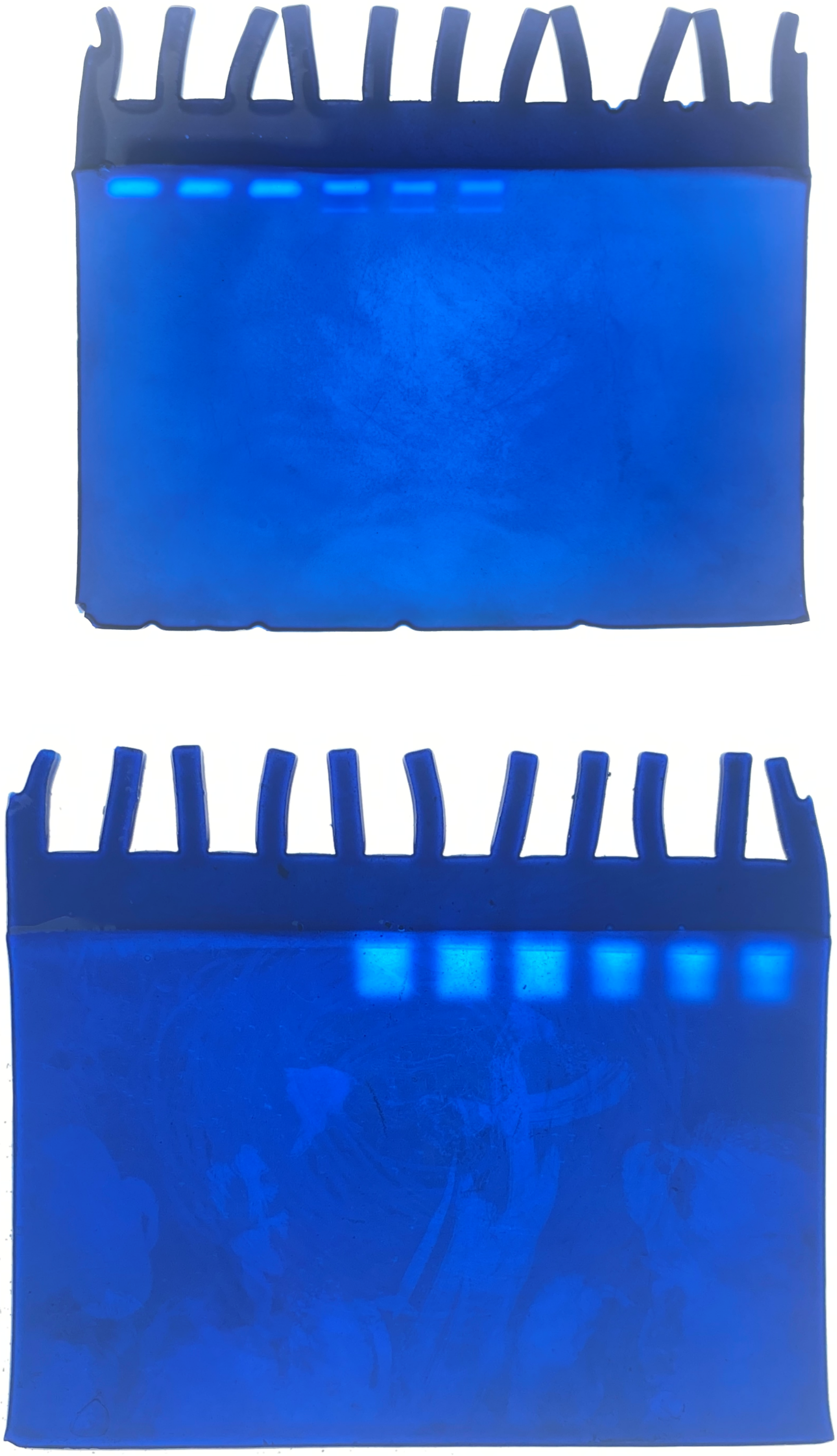

**Figure S1.**. Isozyme profile of CAT from shoots and roots of *P. miliaceum* under treatment with aqueous extract and in control.

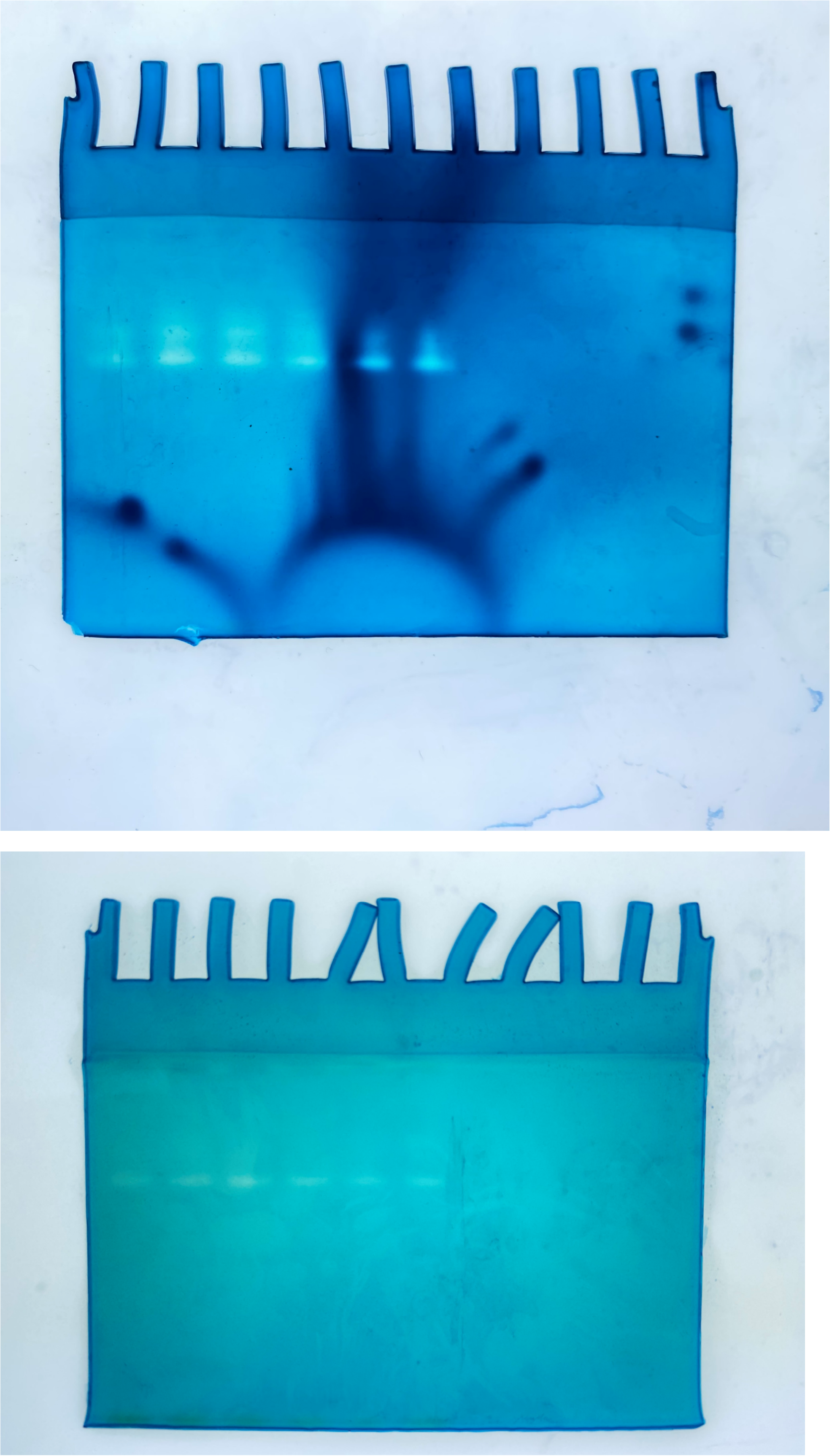

**Figure S2.** Isozyme profile of APX from shoots and roots of *P. miliaceum* under aqueous extract treatment and in control.

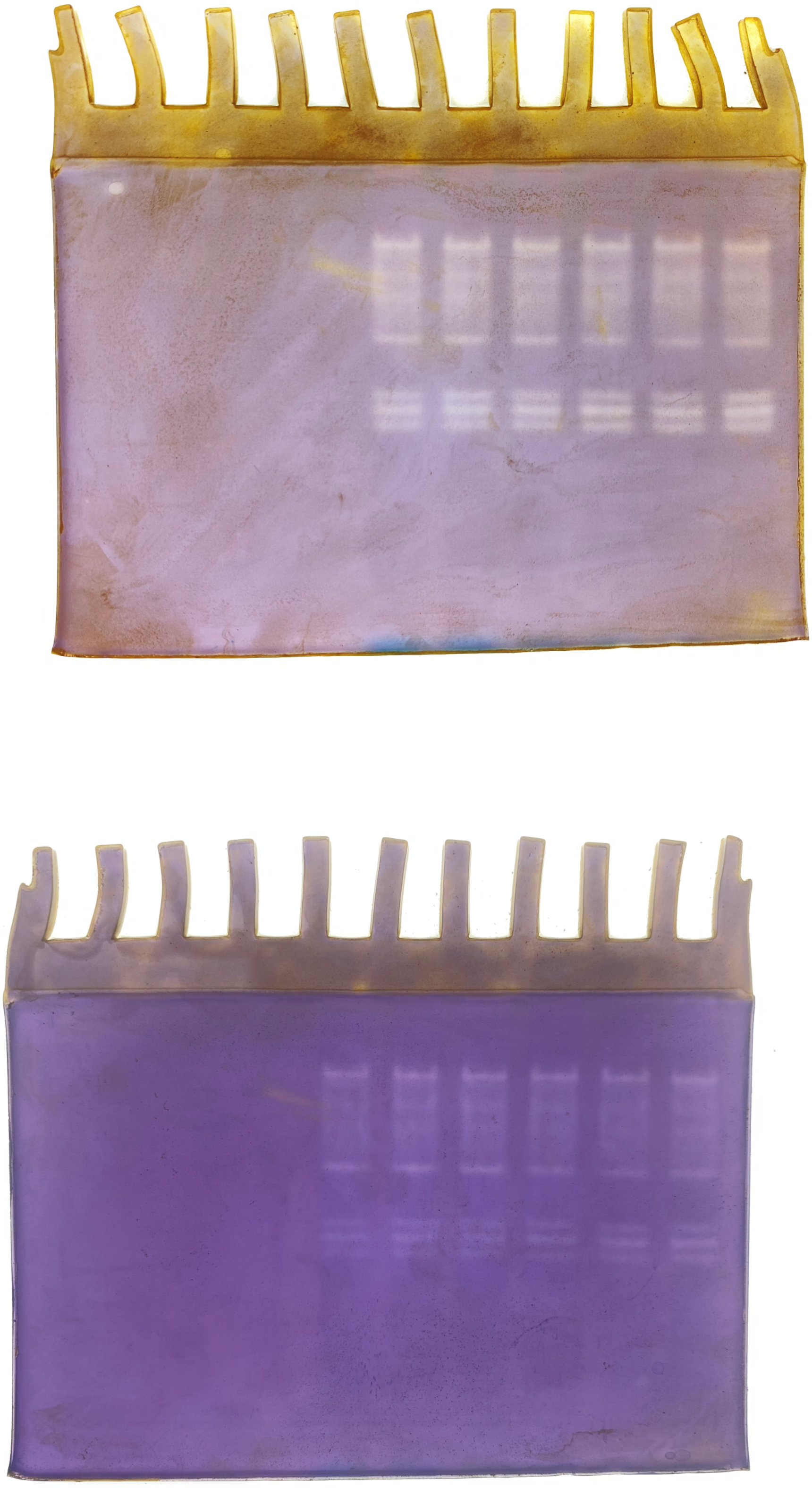

**Figure S3.** Isozyme profile of SOD from shoots and roots of *P. miliaceum* under aqueous extract treatment and in control.

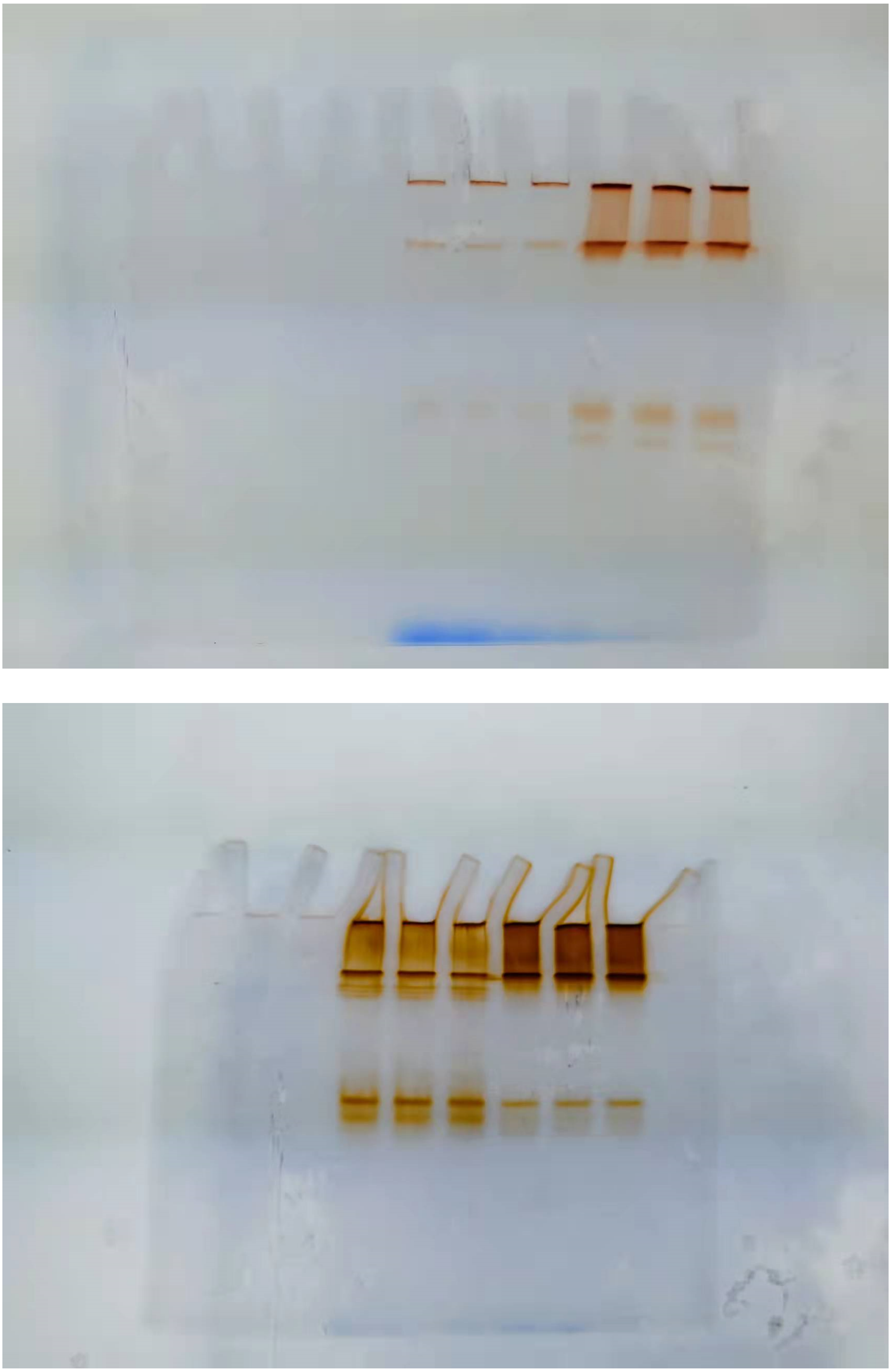

**Figure S4**. Isozyme profile of POD from shoots and roots of *P. miliaceum* under aqueous extract treatment and in control.
